# Projected Non-Monotonic Changes in the Potential Suitable Range of *Phyllanthus emblica* in China Under Future Climate Scenarios

**DOI:** 10.3390/biology15181600

**Published:** 2026-09-11

**Authors:** Yangzhou Xiang, Hongyan Yang, Suhang Li, Qiong Yang, Longcheng Jiang, Wenyuan Chen, Jun Luo, Siyu Zhang, Yinghui Ruan, Chun Ye, Ying Liu

**Affiliations:** 1 School of Geography and Resources, Guizhou Education University, Guiyang 550018, China; 2 Department of Pharmacy, Bijie Medical College, Bijie 551700, China; 3 College of Pharmacy, Guizhou University of Traditional Chinese Medicine, Guiyang 550025, China; 4 School of Biological Sciences, Guizhou Education University, Guiyang 550018, China

**Keywords:** *Phyllanthus emblica*, MaxEnt model, parameter optimization, climate change, suitable habitat zoning

## Abstract

The economically important species *Phyllanthus emblica* L. has received increasing attention, yet its climate-driven distributional shifts across China remain unexplored. Using 446 occurrence records and a parameter-optimized MaxEnt model (RM = 0.1, FC = LQ), we forecasted habitat changes under three SSP scenarios (126, 370, 585) across the 2050s, 2070s, and 2090s. Three temperature variables, namely temperature seasonality (Bio4), temperature annual range (Bio7), and mean temperature of the driest quarter (Bio9), collectively contributed 90.2% to the model, confirming that thermal conditions, particularly during dry seasons, dominate habitat suitability. Current suitable habitat covers 86.43 × 10^4^ km^2^, largely confined to South China’s tropical and southern subtropical belts. Future projections indicate non-linear range responses, with a general northwestward centroid shift, although intermediate periods exhibit oscillatory northeastward and southwestward fluctuations. Under the high-emission SSP585 scenario, modest expansion occurs in high-elevation areas of southeastern Tibet, while contraction affects less than 1% of current suitable low-elevation zones. Based on these findings, we recommend designating long-term core conservation areas (southern Yunnan, southern Guangxi, and Hainan), conducting adaptive introduction trials in expansion zones (e.g., Panzhihua, Sichuan), and implementing planting controls in contraction zones (e.g., northern Guizhou). These insights provide a scientific basis for climate-adaptive management of *P. emblica* in China.

## Simple Summary

Climate change threatens food and medicinal plants by altering where they can grow. We studied *Phyllanthus emblica*, a tropical tree valued for its vitamin-rich fruits, to predict its future habitats across China. Using an optimized computer model with 446 occurrence records and key environmental data, we found that temperature factors, particularly during the dry season, primarily control its distribution, while rainfall is less influential. Currently, the species is projected to be environmentally suitable mainly in southern China. Under future warming scenarios, its suitable range shows a nonlinear response, with a general northwestward shift. Some high-elevation areas may gain suitability, but low-elevation zones face contraction risks. These findings guide conservation and cultivation planning, helping protect wild populations and support sustainable use of this valuable species under climate change.

## 1. Introduction

Global climate change is expected to significantly alter the distribution of plant species worldwide through shifts in temperature, precipitation, and extreme events [1]. Low-temperature stress serves as a key environmental filter defining the northern distribution limit of tropical and subtropical perennial woody plants. Both the mean temperature during the coldest period and the seasonal amplitude of thermal regimes constrain their survival and reproduction [2,3,4]. A common tendency among these species under global warming is the northward and upward shift in their potential ranges. However, contraction or fragmentation may also affect the current ranges of these species [5,6]. Nevertheless, species vary considerably in the direction and magnitude of their response to climate change. This variation depends on multiple interacting factors, including niche breadth, reproductive and dispersal capacity, and the intensity of human disturbance [7,8]. Therefore, conducting fine-scale dynamic assessments of suitable habitats for specific species of economic or ecological importance has become a frontier issue in plant climate geography [9,10]. Species distribution models are the core tool for such research. Among the various species distribution models, MaxEnt is a common choice for simulating potential habitats for crop wild relatives, medicinal plants, and rare tree species. This popularity is largely owing to its high predictive accuracy and robust performance in presence-only data settings [11]. Recent benchmark studies have confirmed MaxEnt’s competitive performance relative to other algorithms [12]. However, traditional MaxEnt modeling often relies on default parameters. This practice easily leads to overfitting or underfitting, which can cause overestimation or underestimation of a species’ potential geographic distribution [13,14]. Recent methodological advances have provided tools for detecting and minimizing overfitting in ecological niche models [15]. Therefore, special attention must be paid to key steps in MaxEnt modeling, including parameter optimization, environmental variable selection, and control of spatial autocorrelation in occurrence points.

*Phyllanthus emblica* L. (*P. emblica*) is an important fruit tree and medicinal plant in the family Phyllanthaceae. Across tropical and subtropical Asia, this species is widespread. Its fruits are rich in vitamin C, polyphenols, and various trace elements. Consequently, it holds broad application prospects in traditional medicine, the food industry, and ecological restoration. Recent years have seen notable progress in studies on its chemical composition [16,17], pharmacological activity [18,19], and genetic diversity of germplasm resources [20,21]. However, research on the spatial response of *P. emblica* to climate change at the national scale remains very limited. In particular, there is a lack of systematic prediction frameworks that integrate multiple scenarios, time periods, and environmental indicators [22]. To date, only one preliminary MaxEnt–ArcGIS study has been conducted on *P. emblica* in China [22], but it relied on limited occurrences and default model parameters. Existing studies are mostly confined to local resource surveys or analyses of single environmental factors. However, with respect to climate, topography, vegetation, and human activity, the drivers of suitable habitat distribution remain poorly understood. This gap severely hampers the development of climate-adaptive management strategies for *P. emblica* and limits its sustainable utilization and conservation planning under climate change.

Environmental variable selection is a core step in species distribution modeling. The relative importance of climatic, topographic, vegetative, and anthropogenic drivers frequently shows considerable variation [23,24]. For tropical and subtropical perennial woody plants, temperature-related factors tend to be more limiting than precipitation-related ones [25,26]. According to previous works, the northward expansion of many tropical fruit trees is primarily driven by three temperature-related variables: mean temperature of the driest quarter, temperature seasonality, and temperature annual range. As a typical tropical and subtropical fruit tree, *P. emblica* is highly sensitive to low-temperature injury. However, it remains unclear both which climatic factors dominate its distribution pattern at the national scale and what the suitable threshold ranges of these factors are, because no quantitative, high-resolution assessment has yet been conducted. Moreover, the human footprint index and the normalized difference vegetation index exert moderating effects that are non-negligible in regional-scale distribution modeling [27,28]. Yet, these indices have rarely been systematically incorporated into previous distribution studies of *P. emblica*. Therefore, it is necessary to apply the variance inflation factor and Spearman correlation analysis for multi-factor screening. This approach avoids multicollinearity while quantifying the relative contributions of different environmental categories [29,30].

The shared socioeconomic pathways (SSPs) within the CMIP6 framework provide a more reliable basis for projecting species distribution dynamics under future climate change [31,32]. Among these, the BCC-CSM2-MR climate model shows strong simulation capabilities for temperature and precipitation in China. It has therefore been widely used in regional-scale ecological response studies [33,34]. This study integrates three SSP scenarios (i.e., SSP126, SSP370, and SSP585) across three future time periods of the 2050s, 2070s, and 2090s. They also cover the time windows in which global mean temperature surpasses the critical thresholds of 1.5 °C and 2.0 °C under different emission scenarios. Analyzing the direction and distance of centroid shifts can quantitatively characterize the dynamic response trajectory of a species’ suitable habitats to climate change [35,36]. Meanwhile, classifying future suitable habitats into stable, contraction, and expansion zones provides an intuitive and quantifiable framework for assessing species distribution dynamics [37]. However, no study to date has systematically evaluated the future distribution dynamics and spatial shifts of *P. emblica* across China by integrating the above multi-scenario, multi-period framework with a parameter-optimized MaxEnt model.

Therefore, comprehensive predictions of the response of *P. emblica* to climate change at the national scale remain lacking. In particular, there is no systematic modeling framework that simultaneously addresses parameter optimization, variable selection, spatial autocorrelation control, multi-scenario and multi-period simulations, and spatial dynamics analysis. To address this gap, the present study uses 446 occurrence points and an optimized MaxEnt model (RM = 0.1, FC = LQ). It incorporates the BCC-CSM2-MR climate model under three SSP scenarios (SSP126, SSP370, and SSP585) across three time periods (2050s, 2070s, and 2090s). The objectives are (1) to predict the potential suitable habitats of *P. emblica* in China under current climatic conditions and to classify them into high, medium, and low suitability levels; (2) to identify the key environmental factors governing its distribution and their response thresholds; (3) to quantify future changes in suitable habitat area under different climate scenarios, including stable, contraction, and expansion zones; and (4) to characterize the migration pathways and directions of the centroid of suitable habitats. These findings provide a decision-making reference for climate-adaptive cultivation planning, germplasm conservation, and sustainable utilization of *P. emblica*.

## 2. Materials and Methods

### 2.1. Acquisition and Processing of Species Distribution Data

In this study, we integrated multi-source data to build a distribution dataset for *P. emblica*. The data were obtained from four sources: the Chinese Virtual Herbarium (CVH, http://www.cvh.org.cn/, accessed on 2 December 2025) and the Global Biodiversity Information Facility (GBIF, https://doi.org/10.15468/dL.8qxrfu, accessed on 3 December 2025). Additionally, to supplement public databases, we conducted a systematic literature search using the China National Knowledge Infrastructure (CNKI, https://www.cnki.net) and Web of Science (WOS, https://www.webofscience.com) with the search terms “(*Phyllanthus emblica* OR *Emblica officinalis*) AND (distribution OR occurrence OR habitat OR location)” in Chinese and English, covering publications from 1980 to 2026. We manually screened titles and abstracts to identify studies reporting geographic coordinates or detailed locality descriptions for *P. emblica* in China. Inclusion criteria required: (1) clear locality description at the village; (2) explicit confirmation of wild populations; and (3) availability of collection date or approximate year. 

**Figure 1:**
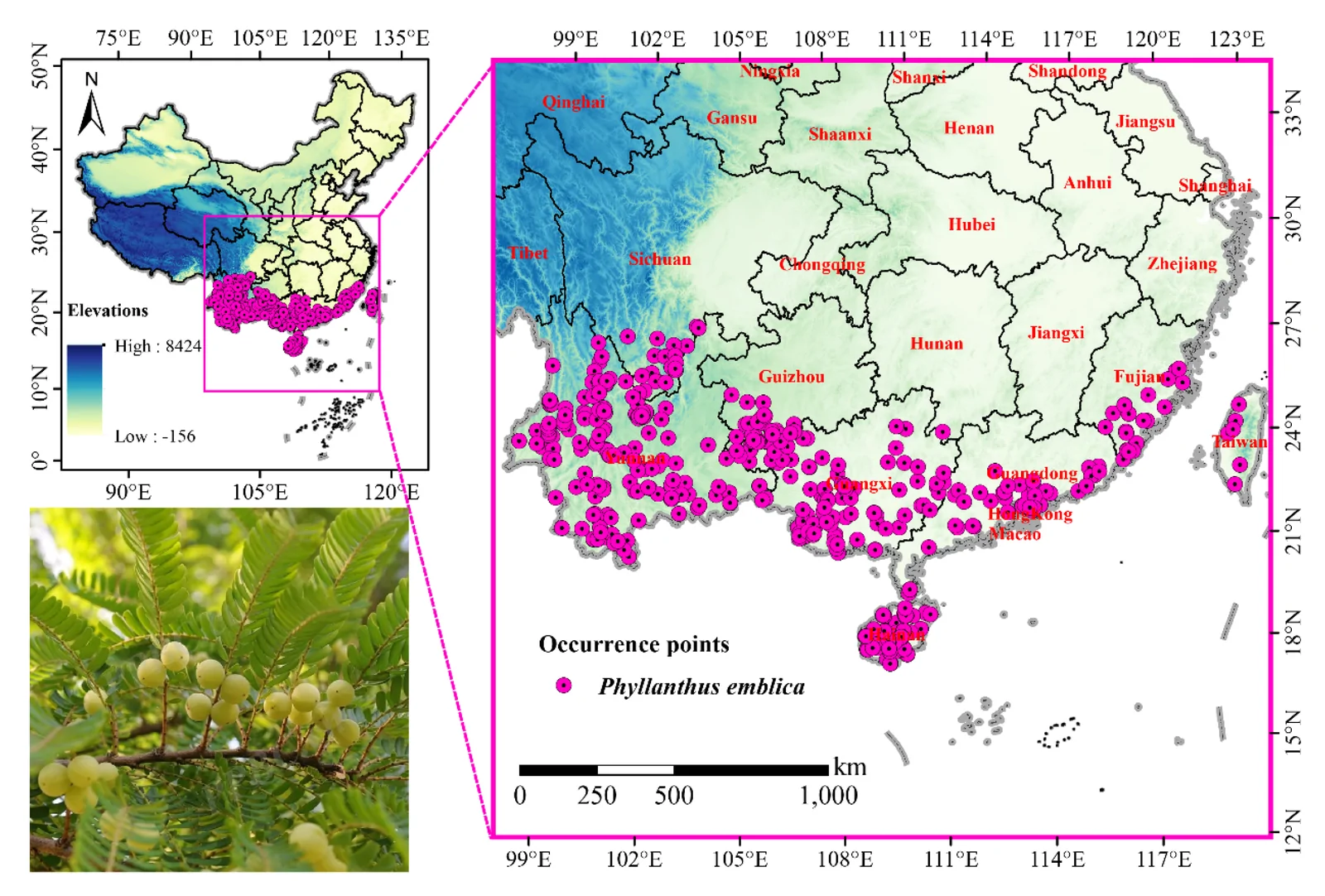
Geographic locations of 446 recorded occurrence sites for *P. emblica* across China. The base map of China for our study was sourced from the Standard Map Service System of the Ministry of Natural Resources of China (Approval No. GS (2023)2762) on 22 December 2023 (http://bzdt.ch.mnr.gov.cn/). The inset photograph of *P. emblica* is reproduced from the Plant Photo Bank of China (PPBC) under a Creative Commons Attribution License (CC BY 4.0) (https://ppbc.iplant.cn/tu/2832394; accessed on 28 August 2026).

We optimized the occurrence points using the following procedure to reduce the impact of spatial autocorrelation on model prediction accuracy. First, we used ArcGIS software (v10.8) to create a 2.5′ × 2.5′ grid covering the study area, which served as the spatial thinning grid. Second, within each grid cell, we retained only the point closest to the cell center. This approach maintains the integrity of the distribution range while minimizing sampling bias caused by point clustering. Third, we saved the final set of 446 occurrence points (240 from GBIF, https://doi.org/10.15468/dl.gzswf9, accessed on 3 December 2025, 104 from CVH, 82 from CNKI, and 20 from WOS; Figure 1) as a CSV file to meet the input requirements of MaxEnt 3.4.4 (http://biodiversityinformatics.amnh.org/open_source/maxent/, accessed on 8 December 2025). This procedure ensures spatial independence of the data and provides a reliable foundation for subsequent species distribution modeling.

### 2.2. Environmental Variable Collection and Selection

In this study, we initially selected 24 candidate environmental variables from four categories (climate, topography, vegetation, and human activity) to build a species distribution model for *P. emblica* under the baseline period (1970–2000) (Table 1). Among these, 19 bioclimatic factors [38] and elevation data were sourced from the WorldClim 2.1 database (https://www.worldclim.org/data/index.html, accessed on 12 December 2025). Subsequently, we derived two topographic variables (slope and aspect) from the elevation data using ArcGIS software (v10.8). Vegetation condition was represented by the normalized difference vegetation index (NDVI) from the MODIS/Terra MOD13A3 product [39], while human activity intensity was measured using the human footprint index [27].

To assess the potential impacts of future climate change on species distribution, we used the BCC-CSM2-MR climate model from the Coupled Model Intercomparison Project Phase 6 (CMIP6). This model has shown strong simulation performance for the China region [33,40]. Using this model, we then projected future distribution patterns under three Shared Socioeconomic Pathways (SSP126, SSP370, and SSP585) across three future time periods: the 2050s (2041–2060), 2070s (2061–2080), and 2090s (2081–2100). Both the native spatial resolution and the final modeling resolution of all environmental variables were 2.5′ × 2.5′. SSP126, SSP370, and SSP585 represent increasing levels of radiative forcing and societal challenges: SSP126 (low forcing, ~2.6 W m^−2^) represents a sustainability scenario with stringent mitigation; SSP370 (medium-high forcing, ~7.0 W m^−2^) represents a regional rivalry scenario with moderate mitigation challenges; and SSP585 (high forcing, ~8.5 W m^−2^) represents a fossil-fueled development scenario with high emissions and no effective mitigation [32].

**Figure 2:**
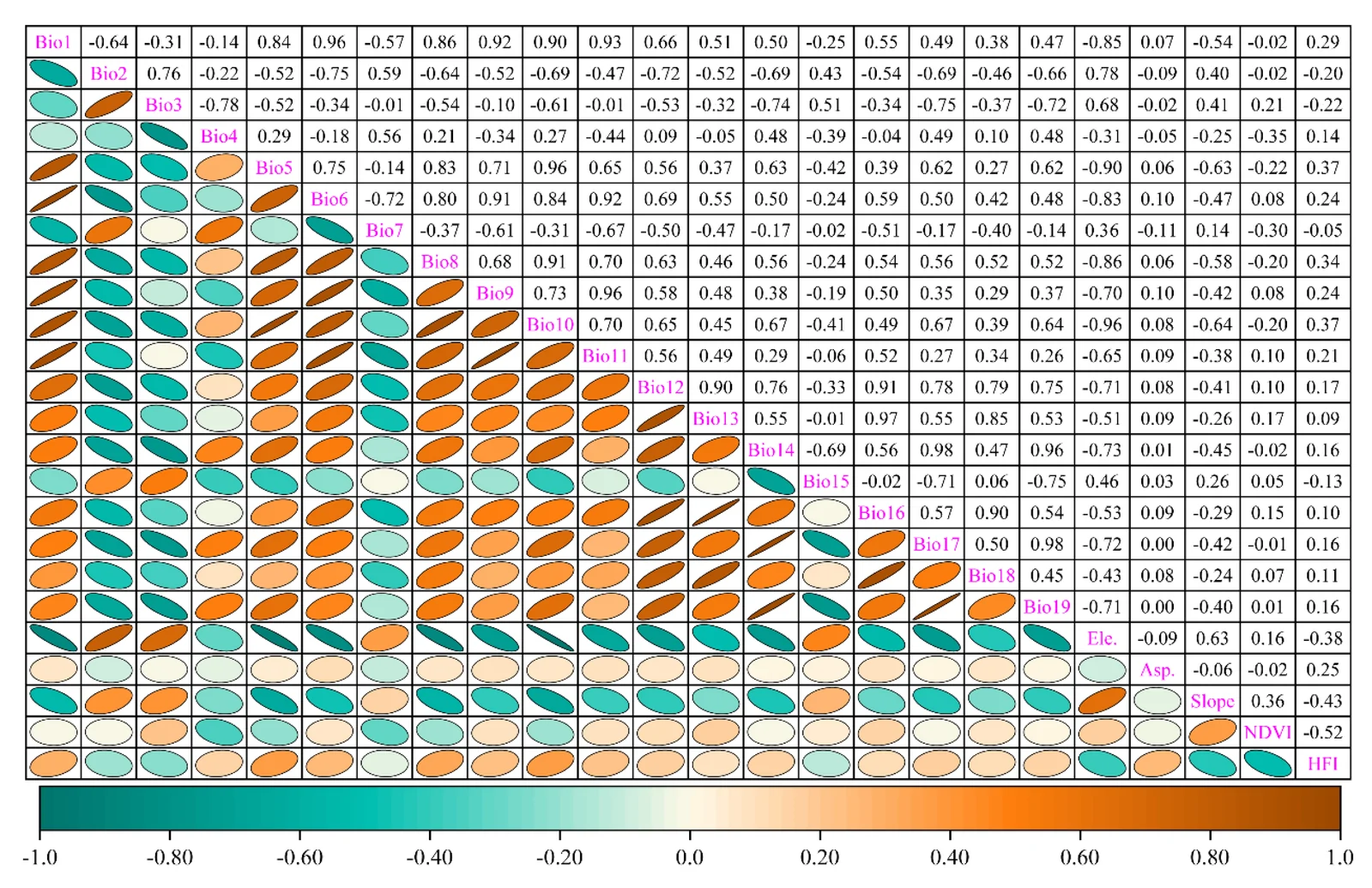
Spearman correlation coefficients among the 24 candidate environmental variables. Flatter brown ellipses represent stronger positive correlations, while flatter cyan ellipses indicate stronger negative correlations. Ele. and Asp. denote elevation and aspect, respectively. Full names and units of all bioclimatic variables (Bio1–Bio19) are provided in Table 1.

To reduce the influence of multicollinearity among environmental factors on model performance and variable interpretability, we calculated the Variance Inflation Factor (VIF) and performed Spearman correlation analysis (Figure 2) using the usdm package (v2.1-7) in R 4.2.2 [41]. Based on the results and applying the thresholds of VIF < 10 and an absolute correlation coefficient < 0.7 [29], we selected environmental factors with low multicollinearity from the 24 candidate variables. This process yielded a final set of 12 variables across four categories: climate (8 variables), topography (2), vegetation (1), and human activity (1) (Table 2).

In the study region (southern China), the driest quarter (typically December–February) coincides spatially and temporally with the coldest quarter for the majority of the occurrence records. To quantitatively verify this overlap, we extracted monthly temperature and precipitation data for all 446 occurrence sites. The mean temperature of the driest quarter (Bio9) and that of the coldest quarter (Bio11) were highly correlated across the occurrence points (Spearman’s r = 0.96, *p* < 0.001). This strong coherence justifies the ecological interpretation of Bio9 as a proxy for winter low-temperature stress in our study system.

### 2.3. Model Parameter Optimization

We selected MaxEnt over other presence-only models (e.g., GLM, GAM, Random Forest, BRT) for four reasons: its suitability for presence-only data [11,13], its robustness with small sample sizes [12,14], its interpretable continuous outputs are widely used in conservation [9,10], and its capacity for systematic parameter optimization to reduce overfitting [14,42]. Given these advantages and its broad application for tropical crop wild relatives in China [43,44], MaxEnt was adopted as the core modeling tool.

To improve the accuracy and robustness of MaxEnt in predicting the potential distribution of *P. emblica*, we systematically optimized two key parameters: feature combination (FC) and regularization multiplier (RM). These parameters jointly control model complexity and generalization ability. Appropriate parameter settings help characterize species–environment relationships more accurately, thereby enhancing both ecological plausibility and spatial accuracy. We performed a grid search using the ENMeval 2.0.4 package in R 4.2.2 [14]. For RM, we tested values from 0.1 to 2.0 at 0.1 increments. For FC, we tested nine combinations (H, HPT, L, LQ, LQH, LQHP, LQHPT, QHP, QHPT) derived from five feature classes: linear (L), quadratic (Q), product (P), threshold (T), and hinge (H) [45]. The optimal parameter configuration was selected based on the minimum corrected Akaike Information Criterion (AICc) [42] and was further confirmed by applying the threshold (Delta AICc ≤ 2) recommended by Anderson and Gonzalez [46].

### 2.4. Model Calibration and Performance Evaluation

To predict the potential suitable distribution of *P. emblica* under climate change scenarios, we developed an optimized MaxEnt modeling workflow using 446 high-quality occurrence points. The procedure consisted of three steps. First, we applied stratified random sampling to partition the data into training (75%) and validation (25%) sets, preserving environmental representativeness and reducing spatial clustering. Second, we configured the model using cross-validation (10,000 background points, 10 replicates, clamping disabled, and extrapolation enabled) sampled within a biologically defined mask encompassing southern China south of 30° N, where *P. emblica* can naturally occur based on its known tropical–subtropical distribution. This mask excludes northern regions that are environmentally unsuitable and prevents artificially inflated model performance. A spatial bias raster, generated via kernel density estimation of occurrence records, was used to weight background selection and reduce sampling bias [12]. A regularization multiplier of 0.1 and an LQ feature combination were used to balance internal robustness, complexity control, and nonlinear response capture. Third, after 10 model runs, we integrated the outputs into a final logistic suitability score raster, which served as the standardized basis for subsequent multi-scenario analysis and spatial mapping.

We evaluated the predictive performance of the optimized MaxEnt model using three complementary metrics: the area under the receiver operating characteristic curve (AUC), the true skill statistic (TSS), and Cohen’s kappa coefficient (Kappa). AUC measures the model’s ability to discriminate between occurrence points and background environments. Its accuracy is classified as follows: 0.5–0.6 indicates invalid prediction; 0.6–0.7, low accuracy; 0.7–0.8, medium accuracy; 0.8–0.9, high accuracy; and 0.9–1.0, excellent accuracy [45]. TSS and Kappa were calculated using the 10-percentile training presence threshold as the cutoff. Since MaxEnt uses presence-background data, we generated 10,000 background points as the ‘absence’ reference for metric calculation, following the approach of Lawson et al. [47]. The reported values are the mean ± SD across 10 cross-validation folds to account for data-partition variability. TSS integrates sensitivity and specificity, thereby correcting for random bias. Its evaluation criteria are as follows: <0.4 denotes poor, 0.4–0.8 denotes good, and >0.8 denotes excellent [48]. Kappa quantifies the agreement between predicted and actual distributions. Generally, the values of <0.4, 0.4–0.75, and >0.75 indicate poor, good, and excellent agreement, respectively [49].

### 2.5. Classification of Suitability Levels

To define the potential distribution range of *P. emblica*, we used the 10-percentile training presence threshold, which excludes the 10% of occurrence records with the most extreme environmental conditions. This threshold effectively controls commission errors (false positives) while keeping omission errors (false negatives) low and is widely considered robust in ecological niche modeling [13]. Based on the mean suitability score (*p*) from 10 model runs, we set 0.0977 as the suitability threshold in ArcGIS software (v10.8). Using the probabilistic characteristics of actual occurrences, we then classified suitability into four levels: non-suitable (*p* < 0.0977), low suitability (0.0977 ≤ *p* < 0.3), medium suitability (0.3 ≤ *p* < 0.5), and high suitability (*p* ≥ 0.5).

We selected the 10-percentile training presence threshold (0.0977) as the primary cutoff for three reasons. First, it effectively controls commission errors (false positives) while keeping omission errors low and is widely considered robust in ecological niche modeling [11,13]. Second, it excludes the 10% of occurrence records with the most extreme environmental conditions, thereby focusing on core suitable habitats. Third, it represents a conservative choice that avoids overestimating suitable areas compared with the minimum training presence threshold, yet is less restrictive than thresholds based on maximized sensitivity-specificity in our dataset. In addition, the boundaries of 0.3 and 0.5 for low, medium, and high suitability were chosen for cartographic and comparative purposes to facilitate visual interpretation and enable direct comparison with prior studies on tropical fruit trees in China [43,50]. These fixed values do not represent ecologically validated categories, and we emphasize that our management recommendations are primarily based on the binary suitable/non-suitable classification (threshold = 0.0977) rather than on finer suitability divisions.

### 2.6. Analysis of Spatial Pattern Dynamics

Under climate change, the spatiotemporal evolution of a species’ distribution range can be decomposed into three spatial change patterns. First, stable areas (retention zones) are those that are suitable both currently and in the future. Second, contraction zones (loss zones) are those that are currently suitable but lose suitability in the future. Third, expansion zones (gain zones) are those that are currently unsuitable but become suitable in the future [35,37]. To quantitatively analyze the response of suitable habitats of *P. emblica* to different climate scenarios, we performed multi-period spatial change analysis using raster-based algebraic operations in ArcGIS software (v10.8), based on the average suitability probability (*p*) from 10 repeated MaxEnt runs. First, we used 0.0977 as the critical threshold and reclassified the continuous suitability rasters for current and future periods into binary distribution maps (1 = suitable, 0 = non-suitable). Second, we applied the following pixel-level raster algebra to identify three change trajectories: a pixel value change from 1 to 1 indicates retention, from 1 to 0 indicates loss, and from 0 to 1 indicates expansion [35,36,51].

Stable = Current × Future

Loss = Current × (1 − Future)

Gain = (1 − Current) × Future

These formulations ensure strict area conservation, such that Current area = Stable + Loss and Future area = Stable + Gain. All calculations were performed at the native raster resolution (2.5′) without vector conversion, avoiding geometry-repair and sliver-polygon artifacts. Area estimates were derived by multiplying pixel counts by the area of a single pixel (accounting for latitudinal variation under the Mollweide equal-area projection). Percentages for stable, loss, and gain areas were calculated with the current suitable area as the denominator [e.g., Stable (%) = Stable/Current area × 100%]. This ensures that the three percentages sum to 100%.

### 2.7. Centroid Displacement Analysis of Suitable Habitats

To quantify the spatial response of *P. emblica*’s suitable habitats to climate change, we characterized their dynamic trajectories using the spatial migration distance and direction of distribution centroids. This analysis was also based on the average suitability probability (*p*) from 10 repeated MaxEnt runs. The analytical procedure involved three steps. First, we applied a threshold of 0.0977 to binarize the probability raster, thereby delineating suitable areas and converting them into vector polygons in ArcGIS software (v10.8). Second, using the geometric center calculation function in the spatial statistics module, we extracted the centroid coordinates of the suitable areas for each scenario and time period. Third, based on the latitudinal and longitudinal changes in the centroid points across spatiotemporal intervals, we calculated the Euclidean distance to represent migration magnitude and determined the migration direction using the azimuth angle. This approach systematically reveals the spatial displacement trends of species distribution centroids under climate change [52,53].

## 3. Results

### 3.1. Model Optimization and Accuracy Assessment

**Figure 3:**
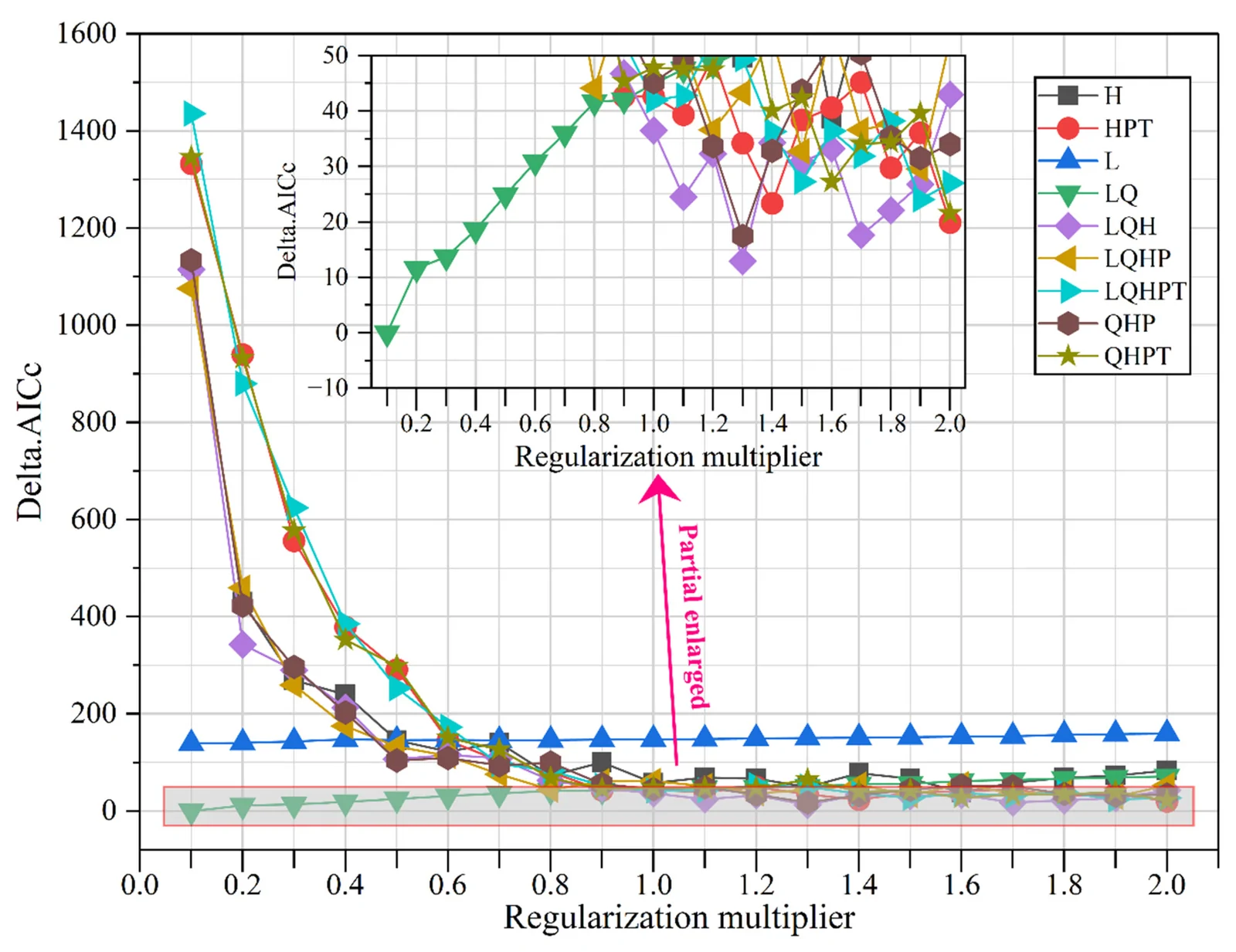
Variation in ΔAICc among different feature class (FC) combinations for *P. emblica*. FC abbreviations: L, Linear; Q, Quadratic; H, Hinge; P, Product; T, Threshold.

**Figure 4:**
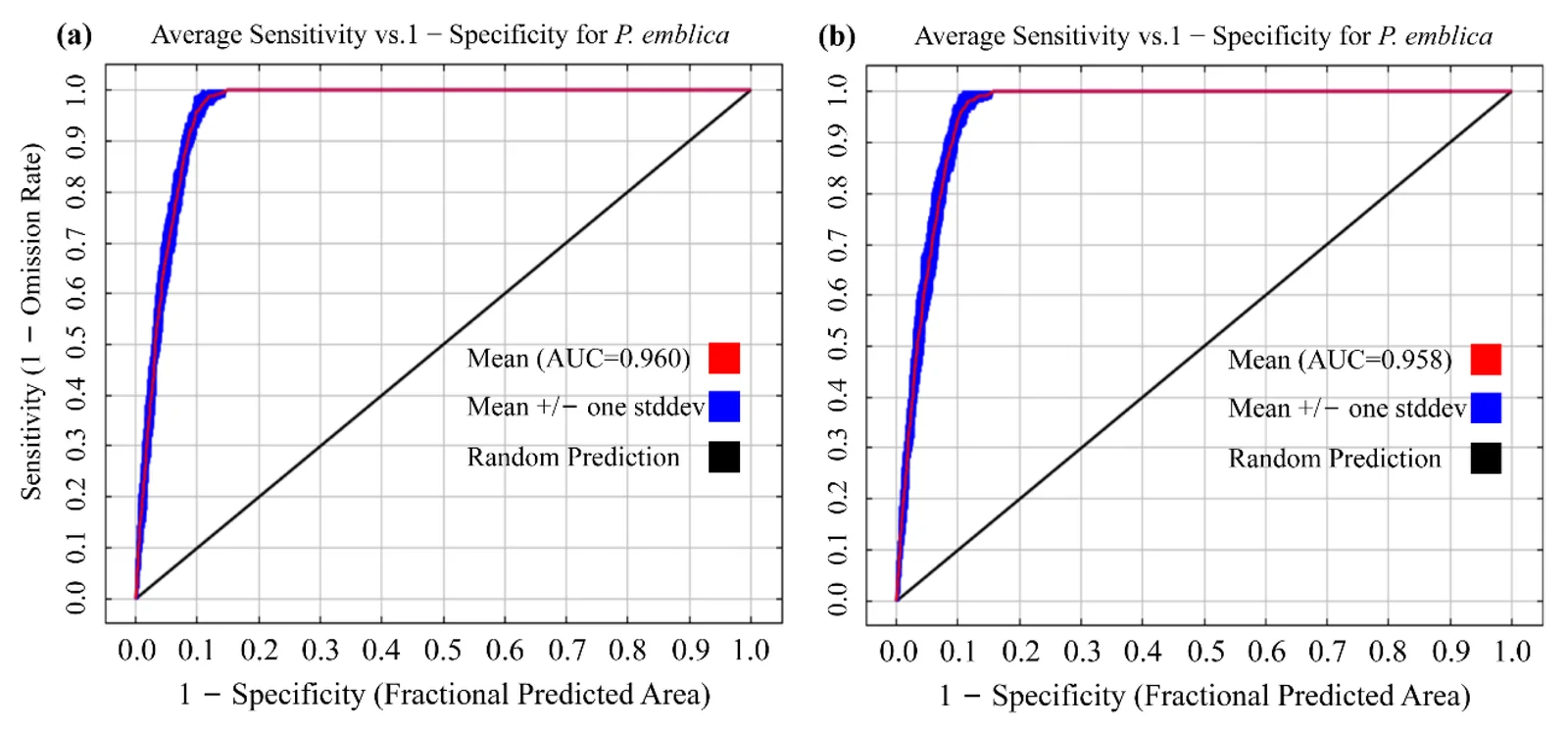
Receiver operating characteristic (ROC) curves illustrating the predictive performance of the *P. emblica* MaxEnt model, with AUC values shown for (**a**) default and (**b**) optimized parameters.

The MaxEnt model for *P. emblica* built with default parameters (RM = 1, FC = LQHP) produced a ΔAICc value of 61.61 (Figure 3), which far exceeds the recommended threshold (ΔAICc ≤ 2), indicating severe overfitting. Through systematic grid search, the optimal parameter combination was identified as RM = 0.1 and FC = LQ, under which the ΔAICc dropped to zero, suggesting an optimal trade-off between data fitting and model complexity. Accuracy assessment (Figure 4) showed that the AUC values before and after optimization were 0.960 (SD = 0.005) and 0.958 (SD = 0.006), respectively, both falling within the excellent range (AUC ≥ 0.9), implying that parameter simplification did not compromise the model’s discriminatory ability. The optimized model achieved a true skill statistic (TSS) of 0.899 (SD = 0.003), reflecting excellent discrimination performance, and a Kappa coefficient of 0.653 (SD = 0.009), which lies within the good agreement range. Together, these three metrics indicate that the optimized model combines high predictive accuracy with strong robustness and can therefore be effectively applied to simulate suitable habitats under future climate change scenarios.

### 3.2. Dominant Environmental Factors

**Figure 5:**
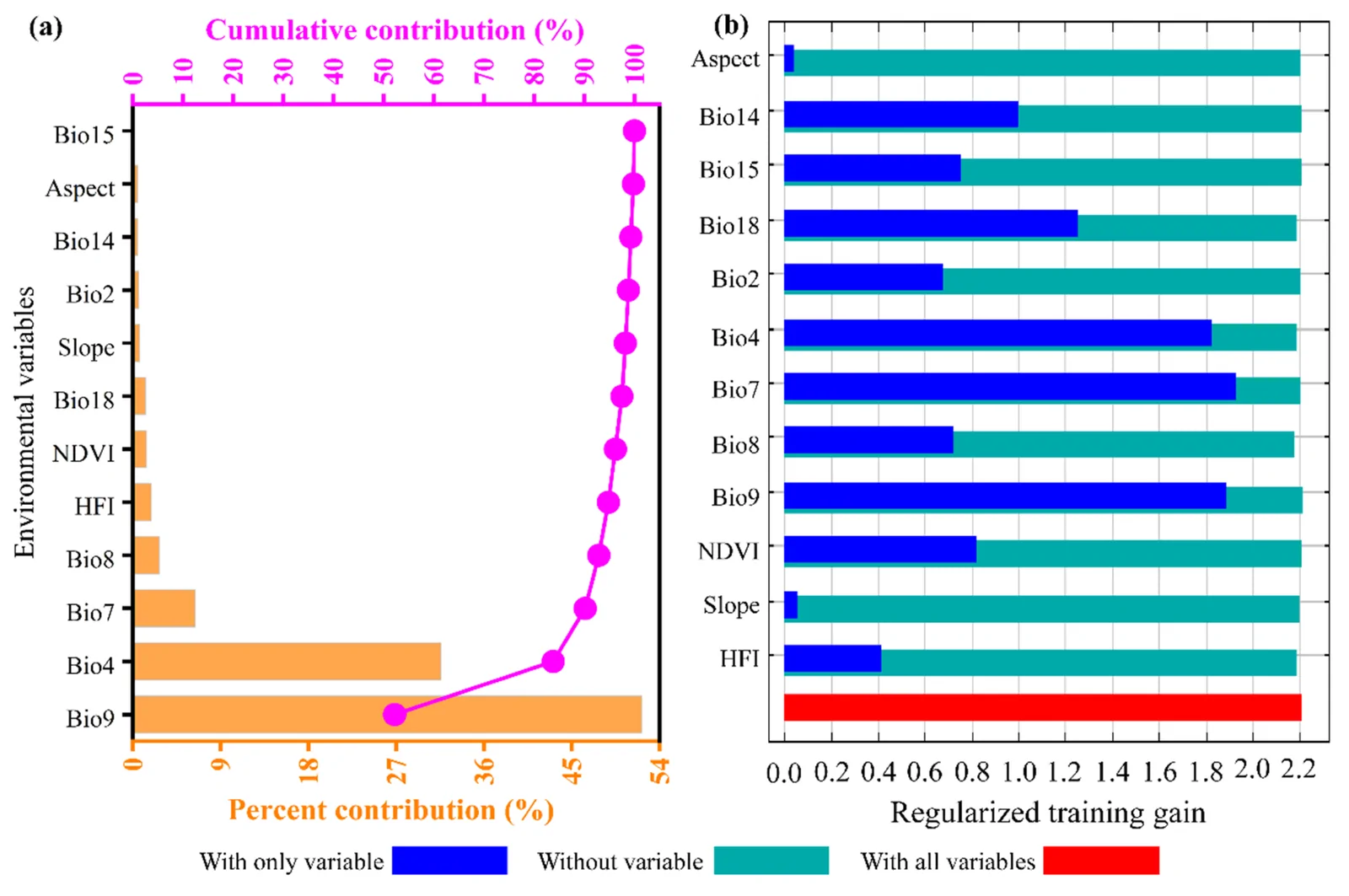
Relative importance of environmental predictors for *P. emblica* in the MaxEnt model: (**a**) percent contribution and (**b**) jackknife test of regularized training gain.

Using the optimized MaxEnt model, we systematically analyzed the influence of 12 environmental factors on the potential geographic distribution of *P. emblica*. This analysis integrated variable contribution rates and jackknife test results. The environmental variables ranked by contribution rate (Figure 5a) are as follows: mean temperature of driest quarter (Bio9, 52.2%), standard deviation of temperature seasonality (Bio4, 31.6%), temperature annual range (Bio5–Bio6) (Bio7, 6.4%), mean temperature of wettest quarter (Bio8, 2.7%), human footprint index (HFI, 1.9%), normalized difference vegetation index (NDVI, 1.4%), precipitation of warmest quarter (Bio18, 1.3%), slope (0.7%), mean diurnal range (mean of monthly) (Bio2, 0.6%), precipitation of driest month (Bio14, 0.5%), aspect (0.5%), and variation in precipitation seasonality (Bio15, 0.2%). By factor category, climatic factors contributed 95.5% in total, followed by human activity (1.9%), vegetation (1.4%), and topography (1.2%). The regularized training gain results from the jackknife test (Figure 5b) further showed that, when used in isolation, the five variables with the highest gain values were Bio9, Bio7, Bio4, Bio18, and NDVI. According to the standard screening criterion of cumulative contribution exceeding 85%, Bio9, Bio4, and Bio7 together explained 90.2% of the variation in suitable habitat for this species. This indicates that these low-temperature-related factors have a decisive influence on the potential geographic distribution of *P. emblica* in China, serving as the core climatic drivers of its distribution pattern.

**Figure 6:**
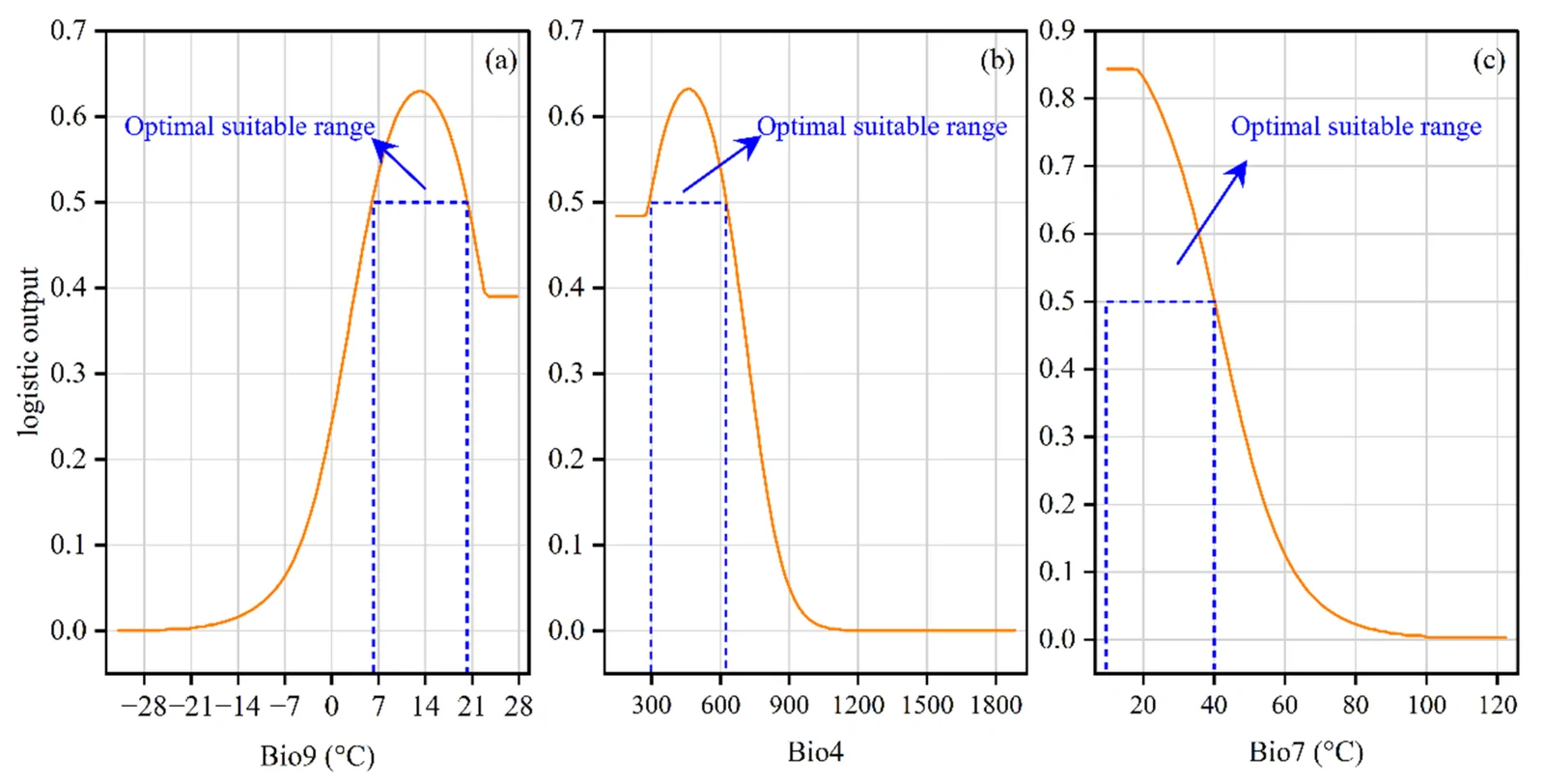
Average response curves of *P. emblica* to key environmental drivers. The orange line indicates the suitability score. The blue dashed lines delimit the model-derived suitable intervals (logistic probability ≥ 0.5). These intervals reflect relative suitability predicted by the model and do not represent independently validated physiological or growth optima. (**a**) Mean temperature of the driest quarter; (**b**) Temperature seasonality; (**c**) Temperature annual range.

Having identified these three dominant environmental factors, we further quantified their independent effects on the distribution probability of *P. emblica* using univariate response curves (Figure 6). The results revealed clear differences in their direction and intensity of influence. For Bio9 (mean temperature of the driest quarter), the response followed a unimodal trend of initial increase followed by a decrease. When the temperature rose from −31.91 °C to −12.38 °C, the suitability probability remained near zero (<0.02). Thereafter, the probability increased rapidly, exceeding 0.20 at −1.17 °C and reaching 0.47 at 5.34 °C. It peaked at 0.63 near 13.92 °C. As the temperature rose further, the probability declined slowly, falling back to 0.39 at 24.19 °C, after which it remained stable within the range of 24.19–27.69 °C. For Bio4 (temperature seasonality), the response curve showed a skewed peak pattern. When the coefficient increased from 146.13 to 290.60, the probability rose slowly from 0.48 to 0.50. It then climbed further to a maximum of 0.63 within the range of 290.61–464.30. Beyond this point, the probability steadily declined, dropping to 0.49 at 634.36 and to only 0.09 at 851.96, eventually approaching zero after exceeding 1075.05. For Bio7 (temperature annual range), the effect on distribution probability was consistently negative. Based on changes in the slope of the curve, this response can be divided into four stages. In the high-probability stable zone (9.85–21.18 °C), the probability decreased only slightly from 0.84 to 0.82. As the temperature range expanded from 21.19 °C to 47.39 °C, the probability entered a rapid decline zone, dropping sharply to 0.34. In the slow decline zone (47.40–57.30 °C), the probability continued to decrease to 0.16. Beyond 57.30 °C, it entered a very low probability zone, falling below 0.16 and remaining stable below 0.04 after 74.30 °C. Using a suitability probability threshold of ≥0.5 to define the model-derived suitable intervals for *P. emblica*, the suitable intervals are as follows: 6.09–20.24 °C for Bio9, 290.59–625.22 for Bio4, and 9.85–39.83 °C for Bio7.

### 3.3. Current Suitable Habitat Distribution

**Figure 7:**
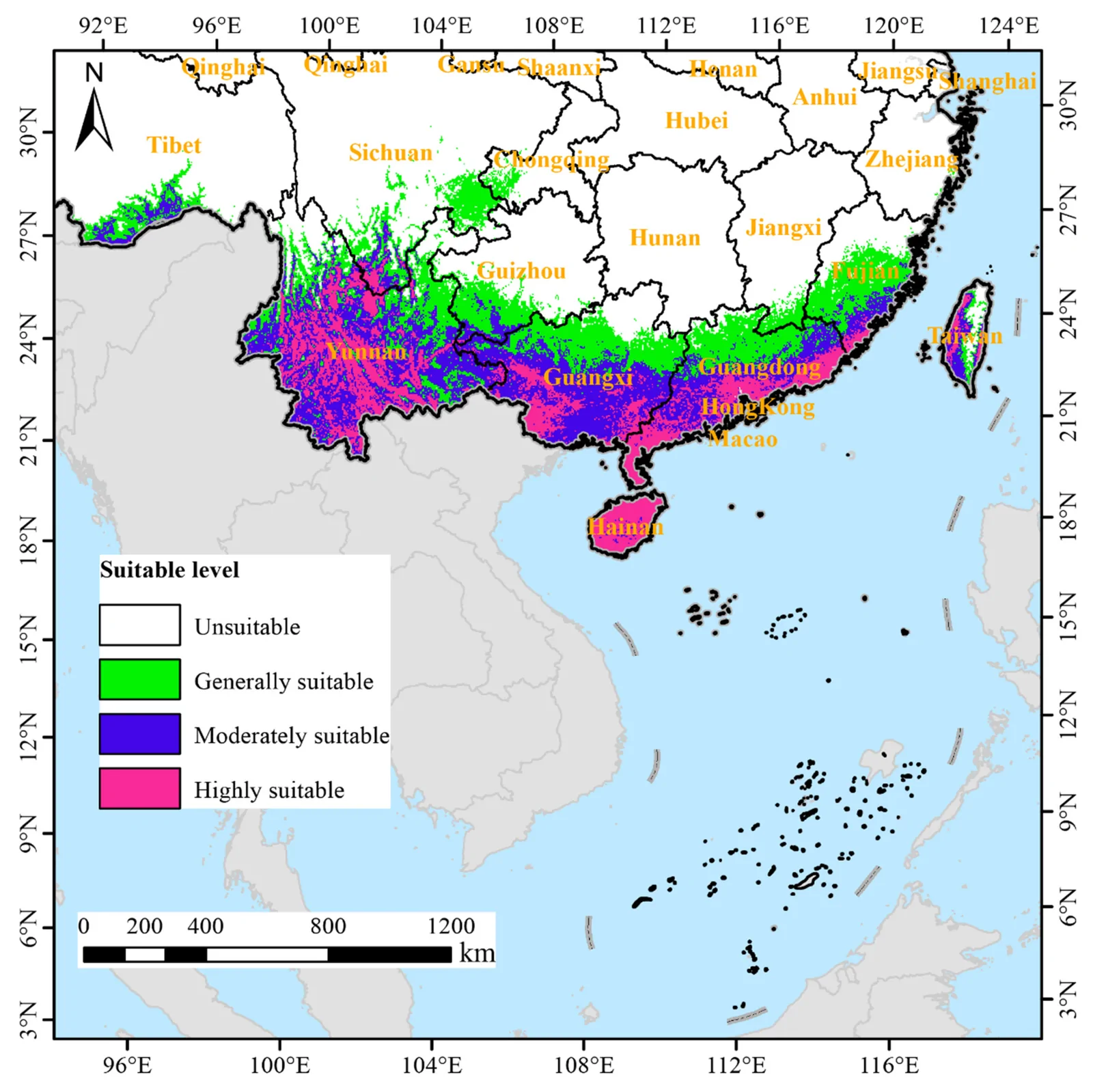
Potential distribution of *P. emblica* under the baseline climate scenario.

Under current climatic conditions, the total potential suitable habitat area for *P. emblica* in China is approximately 86.43 × 10^4^ km^2^, accounting for 9.00% of China’s total land area (Figure 7). Of this, the high-suitability area covers about 21.68 × 10^4^ km^2^ (2.26% of China’s land area) and is mainly concentrated in tropical to southern subtropical regions, including Hainan Province, Yunnan Province, southern Guangxi Zhuang Autonomous Region, southern Guangdong Province, southern Fujian Province, and western Taiwan Province. The medium-suitability area spans approximately 32.30 × 10^4^ km^2^ (3.36%). It primarily covers southern Tibet Autonomous Region, southern Sichuan Province, southern Guizhou Province, eastern Yunnan Province, southern Guangxi Zhuang Autonomous Region, southern Guangdong Province, southern Fujian Province, and western Taiwan Province. The low-suitability area occupies about 32.45 × 10^4^ km^2^ (3.38%). It is distributed across southern Tibet Autonomous Region, northeastern Yunnan Province, eastern Sichuan Province, western Chongqing Municipality, southern Guizhou Province, northern Guangxi Zhuang Autonomous Region, northern Guangdong Province, central Fujian Province, and southern Jiangxi Province.

### 3.4. Future Distribution of P. emblica Under Climate Change Scenarios

**Figure 8:**
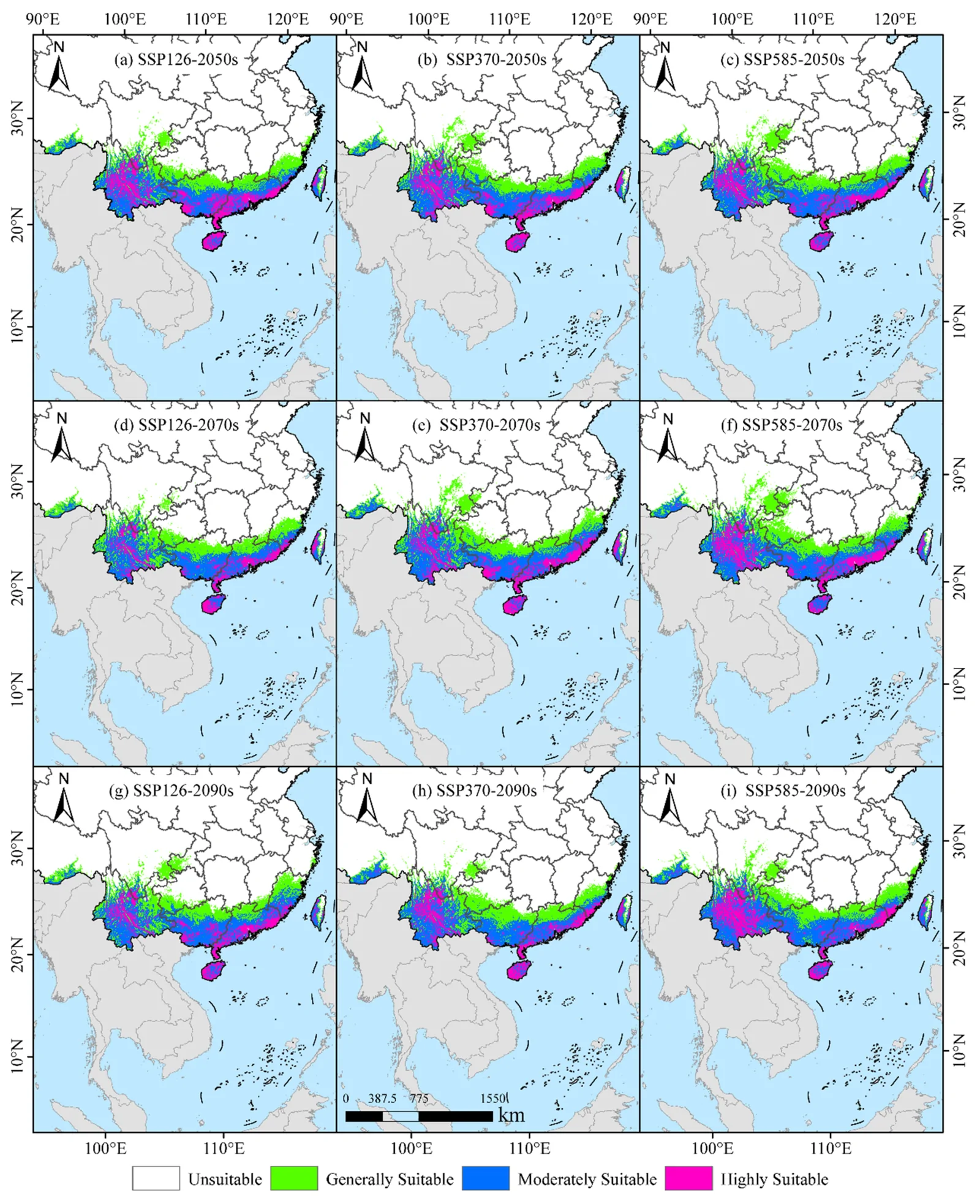
Synthesized projection of suitable habitat for *P. emblica* under three Shared Socioeconomic Pathways (SSP126, SSP370, SSP585) across the 2050s, 2070s, and 2090s.

Under this low-forcing scenario, the total suitable habitat area for *P. emblica* is projected to be 91.24 × 10^4^ km^2^ in the 2050s. It then decreases to 84.73 × 10^4^ km^2^ in the 2070s before rebounding to 94.41 × 10^4^ km^2^ in the 2090s. The corresponding high-suitability areas are 22.26 × 10^4^ km^2^, 13.92 × 10^4^ km^2^, and 17.11 × 10^4^ km^2^, respectively. Spatially, the 2050s suitable areas cover Sichuan Province (Panzhihua City), Yunnan Province (Baoshan City, Chuxiong Prefecture, Dali Prefecture, Honghe Prefecture, Lijiang City, Lincang City, Pu’er City, Wenshan Prefecture, and Yuxi City), Guangxi Zhuang Autonomous Region (Baise City, Beihai City, Chongzuo City, Guigang City, Nanning City, Wuzhou City, and Yulin City), Guangdong Province (Chaozhou City, Dongguan City, Foshan City, Guangzhou City, Huizhou City, Jiangmen City, Jieyang City, Maoming City, Shanwei City, Yangjiang City, Yunfu City, Zhanjiang City, and Zhaoqing City), Fujian Province (Zhangzhou City), Hainan Province, and Taiwan Province. By the 2070s, Panzhihua City (Sichuan Province) becomes unsuitable, whereas Liangshan Prefecture becomes suitable. Additionally, Xishuangbanna Prefecture is added in Yunnan Province, and Quanzhou City is added in Fujian Province. The distribution in other provinces remains largely unchanged from the 2050s (Figure 8d). By the 2090s, Laibin City is added in Guangxi Zhuang Autonomous Region, while Heyuan City and Meizhou City are added in Guangdong Province. In Fujian Province, Longyan City is added. The distribution in Sichuan Province, Yunnan Province, Hainan Province, and Taiwan Province remains the same as in the 2070s (Figure 8g).

Under this medium-high forcing scenario, the total suitable habitat area is projected to be 92.76 × 10^4^ km^2^ in the 2050s, then rises to 97.72 × 10^4^ km^2^ in the 2070s, and finally declines to 91.92 × 10^4^ km^2^ in the 2090s. The corresponding high-suitability areas are 20.93 × 10^4^ km^2^, 19.71 × 10^4^ km^2^, and 16.80 × 10^4^ km^2^, respectively (Figure 8b,e,h). In the 2050s, the suitable areas cover Sichuan Province (Liangshan Prefecture and Panzhihua City), Yunnan Province (Baoshan City, Chuxiong Prefecture, Dali Prefecture, Honghe Prefecture, Kunming City, Lijiang City, Lincang City, Pu’er City, Qujing City, Wenshan Prefecture, Xishuangbanna Prefecture, and Yuxi City), Guangxi Zhuang Autonomous Region (Baise City, Beihai City, Chongzuo City, Guigang City, Nanning City, Wuzhou City, and Yulin City), Guangdong Province (Chaozhou City, Dongguan City, Foshan City, Guangzhou City, Heyuan City, Huizhou City, Jiangmen City, Jieyang City, Maoming City, Meizhou City, Shantou City, Shanwei City, Yangjiang City, Yunfu City, Zhanjiang City, and Zhaoqing City), Fujian Province (Zhangzhou City), Hainan Province, and Taiwan Province. By the 2070s, the distribution expands considerably: Shenzhen City and Zhongshan City are added in Guangdong Province, while Longyan City, Putian City, Xiamen City, and Quanzhou City are added in Fujian Province. The distribution in Yunnan Province and Guangxi Zhuang Autonomous Region remains unchanged from the 2050s (Figure 8e). By the 2090s, Nujiang Prefecture is added in Yunnan Province, whereas Guangxi Zhuang Autonomous Region contracts with the loss of Wuzhou City. Fujian Province retains Zhangzhou City, Putian City, Xiamen City, and Quanzhou City, and Guangdong Province retains its 2070s distribution (Figure 8h).

Under this high-forcing scenario, the total suitable habitat area is projected to be 96.39 × 10^4^ km^2^ in the 2050s, then rises to 100.53 × 10^4^ km^2^ in the 2070s, and reaches 98.50 × 10^4^ km^2^ in the 2090s. The corresponding high-suitability areas are 18.38 × 10^4^ km^2^, 21.20 × 10^4^ km^2^, and 21.30 × 10^4^ km^2^, respectively (Figure 8c,f,i). In the 2050s, the suitable areas cover Sichuan Province (Liangshan Prefecture and Panzhihua City), Yunnan Province (Baoshan City, Chuxiong Prefecture, Dali Prefecture, Honghe Prefecture, Kunming City, Lijiang City, Lincang City, Pu’er City, Nujiang Prefecture, Xishuangbanna Prefecture, and Yuxi City), Guangxi Zhuang Autonomous Region (Baise City, Beihai City, Chongzuo City, Guigang City, Laibin City, Nanning City, Qinzhou City, Wuzhou City, and Yulin City), Guangdong Province (Chaozhou City, Dongguan City, Foshan City, Guangzhou City, Huizhou City, Jiangmen City, Jieyang City, Maoming City, Shantou City, Shanwei City, Shenzhen City, Yangjiang City, Yunfu City, Zhanjiang City, and Zhaoqing City), Fujian Province (Zhangzhou City, Xiamen City, and Quanzhou City), Hainan Province, and Taiwan Province. By the 2070s, Qujing City and Wenshan Prefecture are added in Yunnan Province. In Guangxi Zhuang Autonomous Region, Laibin City remains suitable, but Qinzhou City becomes unsuitable. Zhongshan City is added in Guangdong Province, and Longyan City is added in Fujian Province (Figure 8f). By the 2090s, Wuzhou City in Guangxi Zhuang Autonomous Region becomes unsuitable, while Baise City, Chongzuo City, Guigang City, Nanning City, and Yulin City remain suitable. In Guangdong Province, Zhongshan City and Zhuhai City are added. Fujian Province’s range contracts to only Zhangzhou City, and Yunnan Province retains its 2070s distribution (Figure 8i).

### 3.5. Temporal and Spatial Dynamics of Suitable Habitats for P. emblica Under Future Climate Scenarios

**Figure 9:**
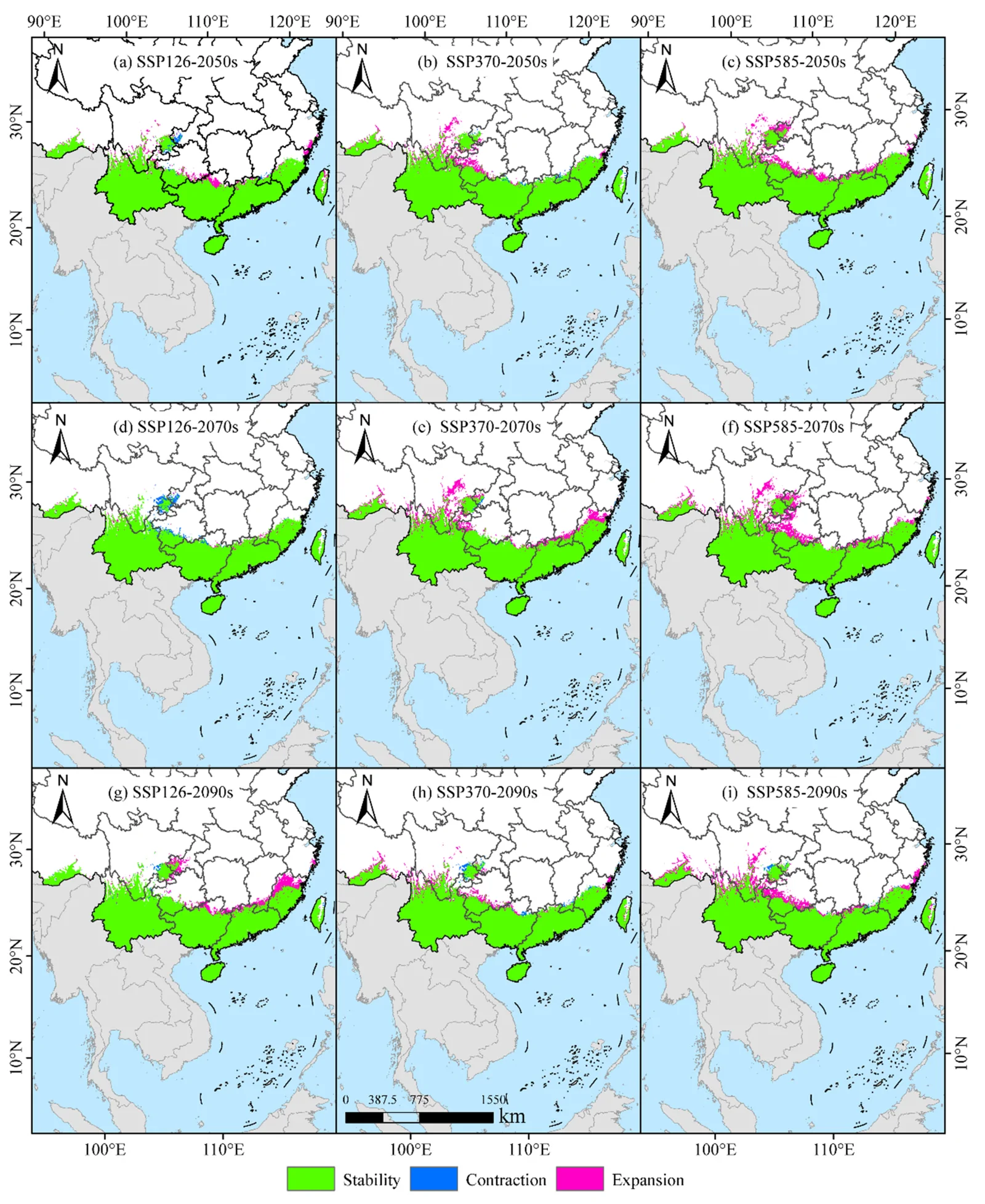
Spatial patterns of distribution stability, contraction, and expansion for *P. emblica* under different climate change scenarios.

A quantitative summary of these area dynamics across all scenarios and periods is presented in Table 3. Under SSP126, the stable, loss, and gain areas in the 2050s are 85.22 × 10^4^ km^2^, 1.21 × 10^4^ km^2^, and 5.91 × 10^4^ km^2^, accounting for 92.28%, 1.31%, and 6.40% of the current suitable area, respectively, with gains mainly distributed across Fujian, Guangdong, Guangxi, Guizhou, Jiangxi, Sichuan, Tibet, Yunnan, Zhejiang, and Taiwan (Figure 9a). By the 2070s, the stable area slightly declines to 83.29 × 10^4^ km^2^, while loss increases to 3.12 × 10^4^ km^2^ and gain shrinks to 1.37 × 10^4^ km^2^ (94.88%, 3.55%, and 1.57%), indicating a notable narrowing of the expansion range (Figure 9d). By the 2090s, the stable area rebounds to 85.75 × 10^4^ km^2^, loss drops to 0.66 × 10^4^ km^2^, and gain increases to 8.59 × 10^4^ km^2^ (90.26%, 0.70%, and 9.04%), with expansion extending northeastward to include Chongqing and Taizhou (Zhejiang) (Figure 9g).

Under SSP370, the stable, loss, and gain areas in the 2050s are 85.64 × 10^4^ km^2^, 0.78 × 10^4^ km^2^, and 7.12 × 10^4^ km^2^ (91.55%, 0.84%, and 7.61%), with gains distributed across Fujian, Guangdong, Guangxi, Guizhou, Jiangxi, Sichuan, Tibet, Yunnan, Chongqing, and Taiwan (Figure 9b). By the 2070s, gain increases significantly to 11.66 × 10^4^ km^2^, while stable rises slightly to 85.91 × 10^4^ km^2^ and loss declines to 0.50 × 10^4^ km^2^ (87.61%, 0.51%, and 11.89%). The expansion extends northward and into the western mountains, adding Hunan and Neijiang (Sichuan) (Figure 9e). By the 2090s, stable decreases to 84.95 × 10^4^ km^2^, loss rebounds to 1.45 × 10^4^ km^2^, and gain is 6.89 × 10^4^ km^2^ (91.06%, 1.56%, and 7.38%), with expansion shifting toward higher elevations and adding Zunyi (Guizhou), Luzhou (Sichuan), and Wenzhou (Zhejiang) (Figure 9h).

Under SSP585, the stable, loss, and gain areas in the 2050s are 86.17 × 10^4^ km^2^, 0.25 × 10^4^ km^2^, and 9.91 × 10^4^ km^2^ (89.45%, 0.26%, and 10.29%), representing the most extensive expansion across all scenarios, covering Fujian, Guangdong, Guangxi, Guizhou, Hunan, Jiangxi, Sichuan, Tibet, Yunnan, Zhejiang, Chongqing, and Taiwan (Figure 9c). By the 2070s, gain reaches its maximum across all scenarios and periods (14.12 × 10^4^ km^2^), with stability at 86.20 × 10^4^ km^2^ and loss at only 0.21 × 10^4^ km^2^ (85.74%, 0.21%, and 14.05%). Newly added areas include Nanping (Fujian), Chenzhou (Hunan), Luzhou, Yibin, Ziyang (Sichuan), and Taizhou (Zhejiang) (Figure 9f). By the 2090s, stable slightly declines to 85.73 × 10^4^ km^2^, loss rises to 0.67 × 10^4^ km^2^, and gain is 12.59 × 10^4^ km^2^ (86.60%, 0.68%, and 12.72%). Notably, expansion appears for the first time in Changdu (Tibet), indicating a shift toward higher elevations, with further expansion in Sichuan and Hunan (Figure 9i).

### 3.6. Centroid Migration of P. emblica Suitable Habitats Under Different Climate Scenarios

**Figure 10:**
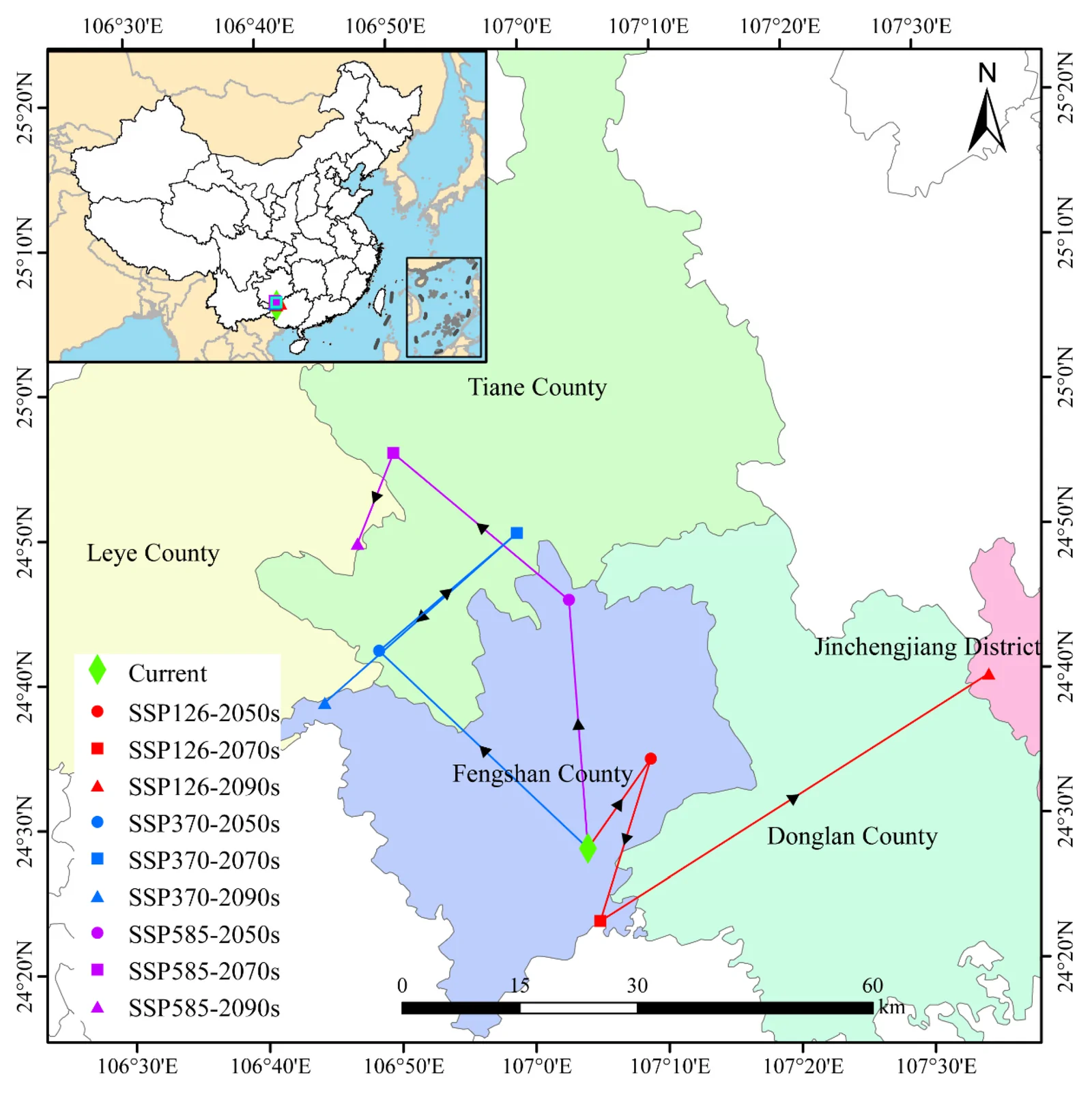
Centroid migration patterns of suitable habitat for *P. emblica* under three SSPs (SSP126, SSP370, SSP585) across the 2050s, 2070s, and 2090s. Centroids are shown at the county-level resolution; village/township names are omitted to avoid false precision due to modeling resolution (2.5′) and the absence of uncertainty envelopes.

Under current climatic conditions, the centroid of the suitable distribution area for *P. emblica* is located at Fengcheng Town, Fengshan County, Hechi City, Guangxi Zhuang Autonomous Region (107°04′ E, 24°28′ N). Under different future climate scenarios, the distribution centroid consistently shows a general trend of northwestward migration (Figure 10). Under the SSP126 scenario, the centroid migrates approximately 13.96 km northeastward from Fengcheng Town to Changzhou Town (107°09′ E, 24°34′ N) in the 2050s, then shifts back southwestward by about 21.76 km to Paoli Township (107°05′ E, 24°23′ N) in the 2070s, and finally migrates substantially northeastward by approximately 58.66 km to Jiuwei Town (107°35′ E, 24°40′ N) by the 2090s. Under the SSP370 scenario, the centroid migrates approximately 36.62 km northwestward to Gengxin Township (106°49′ E, 24°42′ N) in the 2050s, shifts northeastward by about 23.09 km to Bala Yao Ethnic Township (106°59′ E, 24°50′ N) in the 2070s, and then migrates southwestward again by approximately 32.64 km to Jinzhuang Village, Qiaoyin Township (106°45′ E, 24°39′ N) in the 2090s. Under the SSP585 scenario, the centroid migrates approximately 31.89 km northwestward to Batong Mountain, Qiaoyin Township (107°03′ E, 24°45′ N) in the 2050s, continues to migrate northwestward by about 29.18 km to Xiangyang Town (106°50′ E, 24°56′ N) in the 2070s, and then shifts southwestward by approximately 12.41 km to Liupai Town (106°47′ E, 24°50′ N) in the 2090s. All distances reported above represent stepwise displacements between consecutive periods. For net displacements from the current baseline and cumulative path lengths. Because the modeling resolution is 2.5′ (~4.6 km at the equator), centroid positions are reported at county-level precision; village-level names are therefore omitted to avoid false precision.

## 4. Discussion

### 4.1. Model Optimization and Performance Assessment for P. emblica

Through systematic parameter optimization, this study confirms that the default MaxEnt settings (RM = 1, FC = LQHP) carry a significant risk of overfitting (ΔAICc = 61.61). In contrast, the optimized combination (RM = 0.1, FC = LQ) achieves an optimal balance between model parsimony and predictive accuracy. This finding aligns with several recent studies, demonstrating that localized parameter tuning is a prerequisite for improving the reliability of species distribution models, particularly when sample sizes are limited or spatial autocorrelation is pronounced [12,14]. Notably, while the AUC value of the optimized model (0.958) is nearly identical to that of the default model (0.960), the sharp decline in AICc indicates that excessive model complexity does not substantially enhance discriminatory power. Instead, it introduces noise fitting [15,54]. Furthermore, the grid-based spatial rarefaction strategy effectively mitigated sampling bias. This approach has recently been shown to significantly improve model transferability in studies predicting the distribution of medicinal plants [55,56]. Although this study focuses on *P. emblica*, the parameter optimization and spatial rarefaction strategies employed offer clear guidance for MaxEnt modeling of other tropical and subtropical cash crops at similar latitudes.

Although the high AUC values (≥0.958) suggest strong discriminatory ability, this result should be interpreted with caution. AUC can be influenced by the geographic extent of the background, and high values may partly reflect the pronounced environmental distinction between occupied and unoccupied regions in southern China. However, our use of spatial block cross-validation and multiple evaluation metrics (TSS and Kappa) provides a more robust performance assessment.

### 4.2. Dominant Environmental Drivers of Suitable Habitat for P. emblica

Both variable contribution rates and jackknife test results consistently indicate that low-temperature-related factors (Bio9, Bio4, Bio7) collectively explain 90.2% of the variation in *P. emblica* distribution. This finding is consistent with the hypothesis that low temperatures primarily constrain the northern distribution limit of tropical woody plants [57,58]. The mean temperature of the driest quarter (Bio9) ranks first, with a contribution rate of 52.2%. In our study region, the driest quarter coincides with the coldest season (winter), so the response curve of Bio9 effectively reflects the species’ sensitivity to winter cold, with a suitable threshold of 6.09–20.24 °C. This range precisely corresponds to the climatic transition zone from the northern tropical margin to the southern subtropical zone in China. Recent studies on other tropical cash crops, such as longan, mango, and macadamia, have identified similar low-temperature limitation patterns. These findings further reinforce the view that winter cold serves as a core environmental filter for the northward expansion of tropical fruit trees [50,59,60]. From a physiological perspective, low temperatures primarily restrict the winter survival of tropical plants by damaging the electron transport chain of photosystem II and inducing the accumulation of reactive oxygen species [61].

In contrast, precipitation-related factors collectively contribute less than 3%. This suggests that water availability is not the primary limiting factor for *P. emblica* distribution at the current national scale. This finding is consistent with the species’ ecological adaptation to the Asian tropical monsoon region. However, the contribution of HFI to the model was relatively low (1.9%). Although its response curve showed a near-linear relationship with habitat suitability, the low heuristic contribution and the possibility that HFI may partly capture accessibility or collection bias rather than purely anthropogenic pressure mean that no strong causal inference should be drawn from this metric alone. We therefore refrain from attributing distributional changes directly to urban expansion or agricultural reclamation based on the present model. Additionally, the relatively low contribution of topographic factors may be because their role in mediating cold air accumulation is already captured by temperature variables. This pattern reflects a hierarchical control observed in studies of thermophilic plant distributions, where temperature plays a dominant role and topography a synergistic one [62,63].

### 4.3. Climate-Induced Shifts in the Potential Distribution of P. emblica

Our results show that the total suitable area for *P. emblica* follows a non-monotonic response trajectory under different SSP scenarios and time periods. Specifically, it initially expands, then fluctuates or contracts. A notable contraction occurs during the 2070s under the SSP126 scenario. This nonlinear response closely resembles predictions for other tropical cash crops, such as mango, wampee, and litchi. This similarity suggests that under low-emission scenarios, early climate warming may promote species expansion toward higher latitudes. However, as temperatures continue to rise beyond critical physiological thresholds, the southern edge of the original suitable range may be gradually lost due to heat stress [43,44,50]. Of particular interest is the first emergence of expansion areas in Changdu Municipality, Tibet Autonomous Region, during the 2090s under the SSP585 scenario. This finding implies that the potential distribution of *P. emblica* may advance into high-elevation river valleys. This observation aligns with the temperature-limitation release hypothesis [64]. However, because species distribution models cannot directly identify causal mechanisms, we cannot rule out synergistic effects from other environmental factors, such as precipitation changes, reduced human activity, or vegetation feedbacks. Importantly, high-elevation expansion often coincides with the contraction of suitable areas at lower elevations. This dynamic may lead to an elevational lift type of distributional reorganization. Similar patterns have been observed in floristic studies on the eastern slope of the Himalayas [65,66].

The centroid shift analysis reveals a general northwestward migration trend of the suitable habitat centroid for *P. emblica* under all scenarios. Nevertheless, notable back-and-forth fluctuations occur during the mid-term period. This finding diverges from the simplified expectation of unidirectional poleward migration. Instead, it aligns more closely with the hypothesis of oscillatory boundary shifts driven by climate variability. Specifically, interannual variability of the East Asian summer monsoon at regional scales may lead to short-term oscillations rather than linear shifts in species distribution centers [1]. From a conservation biology perspective, the northwestern part of Guangxi Zhuang Autonomous Region consistently serves as the core area of centroid activity. This suggests that this region may act as a long-term climate refuge for *P. emblica*. This area features both complex karst landforms and high habitat heterogeneity, which can buffer the direct impacts of climate warming. This result is consistent with the emphasis on topographic complexity in global climate refuge studies [67].

Beyond the environmental drivers included in our model, climate change itself remains an incompletely understood process, and future trajectories are inherently uncertain, which therefore motivated our use of multiple SSP scenarios [31,32]. However, these scenarios do not capture all potential climatic surprises, such as abrupt tipping points or nonlinear circulation shifts. Moreover, biological responses to climate change will be mediated by indirect factors not included in our model, including altered pest and fungal dynamics, wildfires, and shifting biotic interactions such as competition, herbivory, and pollination [68,69,70]. These pathways are rarely integrated into SDMs, yet they may critically influence the realized responses of *P. emblica*. We therefore emphasize that our projections represent potential climatic suitability rather than comprehensive forecasts of future distributions. Species distributions also reflect the interplay between genetic structure and environmental factors, as phenotypic traits are shaped by genotype–environment interactions [71,72]. For *P. emblica*, population genetic differentiation across Yunnan river valleys suggests local adaptation may exist [21], potentially leading to differential climate responses among populations. Interactions among temperature, water, and soil nutrients can produce non-additive effects that correlative models like MaxEnt cannot capture. Our projections should therefore be validated through common garden and transplant experiments.

### 4.4. Prioritizing Core Refugia and Risk-Based Cultivation Zoning for P. emblica

Based on stable area identification derived from multi-scenario future predictions, we propose designating southern Yunnan Province, southern Guangxi Zhuang Autonomous Region, and Hainan Province as priority in situ conservation areas for core germplasm resources. These areas maintain high suitability under all future scenarios and lie within the genetic diversity center of *P. emblica*. Therefore, their conservation priority should be higher than that of marginal populations [73,74]. This strategy aligns closely with the priority-setting principles for global crop wild relative conservation, which prioritize geographic units that are both highly stable under climate change and harbor high genetic uniqueness [75,76]. At the same time, although expansion areas show increased future suitability, they represent novel climatic territories that may lack co-adapted pollinator insects and soil microbial communities. Such areas include Panzhihua City and Liangshan Prefecture in Sichuan Province, as well as northern Fujian Province [68]. Consequently, local adaptation risk assessments should be conducted prior to introduction [69,70]. Accordingly, we recommend establishing relevant research projects within forestry development plans to investigate climate-adaptive introduction technologies for *P. emblica*.

For climate-adaptive cultivation planning, we recommend a management strategy that stabilizes cultivation in the south while expanding it in the north, with region-specific measures. Specifically, in stable areas such as southern Guangxi Zhuang Autonomous Region, southern Yunnan Province, and Hainan Province, efforts should focus on clonal selection of superior germplasm and the promotion of standardized planting. In expansion-contraction transition zones, such as north-central Fujian Province, northern Guangdong Province, and southern Jiangxi Province, a phased introduction process should be implemented. This process should progress from risk assessment to small-scale trials, then pilot-scale tests, and finally large-scale promotion. Contraction areas, including northern Guizhou Province, eastern Sichuan Province, and western Chongqing Municipality, coincide with the current marginal zones of *P. emblica* cultivation. As the probability of suitable habitat continues to decline in these areas under future climate scenarios, further cultivation expansion would face a high risk of yield reduction [77]. Moreover, the human footprint index is the fifth most important contributing variable, with a contribution rate of 1.9%. This indicates that urban expansion and agricultural reclamation have already placed considerable pressure on *P. emblica* habitats [27,78]. Consequently, we recommend including wild populations of *P. emblica* in the revised list of key protected wild plants. We also recommend establishing a dynamic monitoring and early warning platform based on the MaxEnt model.

### 4.5. Study Limitations and Future Directions

(1) While calibrating with Chinese occurrences only may not capture the species’ full Asian niche, this approach is appropriate for our northern-range-limit inference, yet future studies incorporating full-range Asian data would improve global-scale projections. (2) Although spatial block cross-validation reduces overoptimistic performance estimates, residual spatial autocorrelation may persist and should be addressed in future studies using alternative spatially explicit validation frameworks. (3) All future projections rely on a single GCM (BCC-CSM2-MR), which, despite its validated performance over China, cannot fully capture climate model uncertainty, and thus future work should employ multi-GCM ensembles to improve projection robustness. (4) Environmental novelty analyses (e.g., MESS or MOP) were not conducted, so predictions in high-elevation regions such as southeastern Tibet should be interpreted with caution and validated by future field experiments. (5) The elevational shift patterns reported here are based on a single GCM and threshold, and their consistency across alternative GCMs and threshold choices requires further investigation. (6) MaxEnt predicts potential suitability, not actual establishment or performance, so all management recommendations require field verification. (7) Our projections omit soils, biotic interactions, dispersal, genetic variation, and future land-use change; occurrence records may include cultivated individuals, the equilibrium assumption may not hold, and static NDVI/HFI layers do not capture dynamic feedbacks, while field validation is currently lacking.

## 5. Conclusions

Based on an optimized MaxEnt model, this study identifies the mean temperature of the driest quarter (Bio9), along with temperature seasonality (Bio4) and temperature annual range (Bio7), as the core environmental barriers shaping the distribution of *P. emblica* in China, with a cumulative contribution rate of 90.2%. The current suitable habitat area is approximately 86.43 × 10^4^ km^2^, accounting for 9.00% of China’s land area. This area is primarily concentrated in the tropical to southern subtropical regions of South China. Under future climate scenarios, the suitable habitat exhibits a nonlinear response. The distribution centroid generally shifts northwestward but with mid-term oscillatory fluctuations. Under the high-emission scenario (SSP585), high-elevation expansion occurs in southeastern Tibet, while low-elevation suitable areas face localized contraction risks, though the absolute area remains small.

Based on these findings, we propose concrete management actions: in core stable areas (southern Yunnan, southern Guangxi, Hainan), establish in situ conservation reserves and conduct genetic resource surveys; in expansion zones (e.g., Panzhihua), conduct controlled introduction trials using cold-tolerant genotypes; in contraction zones (e.g., northern Guizhou), develop drought- and heat-tolerant varieties through breeding, evaluate species substitution where cultivation becomes unviable, and consider assisted migration to sites projected to remain suitable. All recommendations are hypothesis-generating and require field validation. This study provides a scientific basis for climate-adaptive cultivation planning and germplasm resource conservation of *P. emblica*.

## Figures and Tables

**Table 1:** Twenty-four environmental variables used in this study.

Category	Abbreviation	Environmental Variables	Units	Range
Bioclimatic	Bio1	Annual mean temperature	°C	10.67–25.85
	Bio2	Mean diurnal range (Mean of monthly)	°C	6.00–12.65
	Bio3	Isothermality (Bio2/Bio7) (×100)		27.49–53.80
	Bio4	Temperature Seasonality (standard deviation ×100)		279.83–746.93
	Bio5	Max temperature of warmest month	°C	21.28–33.48
	Bio6	Min temperature of coldest month	°C	−3.66–17.77
	Bio7	Temperature annual range (Bio5–Bio6)	°C	14.41–27.99
	Bio8	Mean temperature of wettest quarter	°C	16.62–28.96
	Bio9	Mean temperature of driest quarter	°C	3.78–22.85
	Bio10	Mean temperature of warmest quarter	°C	16.62–28.99
	Bio11	Mean temperature of coldest quarter	°C	3.78–21.99
	Bio12	Annual precipitation	mm	640.00–2875.00
	Bio13	Precipitation of wettest month	mm	133.00–777.00
	Bio14	Precipitation of driest month	mm	4.00–56.00
	Bio15	Precipitation Seasonality (Coefficient of Variation)		45.08–112.05
	Bio16	Precipitation of wettest quarter	mm	362.00–1889.00
	Bio17	Precipitation of driest quarter	mm	14.00–199.00
	Bio18	Precipitation of warmest quarter	mm	299.00–1889.00
	Bio19	Precipitation of coldest quarter	mm	15.00–284.00
Topographic	Altitude	Altitude	m	4.00–2500.00
	Aspect	Aspect	°	0.34–358.01
	Slope	Slope	°	0.02–9.25
Vegetation	NDVI	Normalized difference vegetation index		0.14–0.82
Human	HFI	Human footprint index		2.37–47.64

**Table 2:** Environmental predictors selected for the *P. emblica* potential distribution modeling.

Category	Abbreviation	Environmental Variables	Units	VIF
Bioclimatic	Bio2	Mean diurnal range (Mean of monthly)	°C	6.58
	Bio4	Temperature Seasonality (standard deviation ×100)		8.62
	Bio7	Temperature annual range (Bio5-Bio6)	°C	8.29
	Bio8	Mean temperature of wettest quarter	°C	4.67
	Bio9	Mean temperature of driest quarter	°C	5.41
	Bio14	Precipitation of driest month	mm	6.49
	Bio15	Precipitation Seasonality (Coefficient of Variation)		3.16
	Bio18	Precipitation of warmest quarter	mm	2.84
Topographic	Aspect	Aspect	°	1.10
	Slope	Slope	°	1.90
Vegetation	NDVI	Normalized difference vegetation index		1.88
Human	HFI	Human footprint index		1.72

**Table 3:** Projected area dynamics of *P. emblica* suitable habitat under different scenarios.

Scenario-Period	Area (×10^4^ km^2^)			Rate of Change (%)		
	Stability	Contraction	Expansion	Stability	Contraction	Expansion
SSP126-2050s	85.22	1.21	5.91	92.28	1.31	6.40
SSP126-2070s	83.29	3.12	1.37	94.88	3.55	1.57
SSP126-2090s	85.75	0.66	8.59	90.26	0.70	9.04
SSP370-2050s	85.64	0.78	7.12	91.55	0.84	7.61
SSP370-2070s	85.91	0.50	11.66	87.61	0.51	11.89
SSP370-2090s	84.95	1.45	6.89	91.06	1.56	7.38
SSP585-2050s	86.17	0.25	9.91	89.45	0.26	10.29
SSP585-2070s	86.20	0.21	14.12	85.74	0.21	14.05
SSP585-2090s	85.73	0.67	12.59	86.60	0.68	12.72

## Data Availability

The original contributions presented in this study are included in this article. Further inquiries can be directed to the corresponding authors.
